# GrimAge and GrimAge2 Age Acceleration effectively predict mortality risk: a retrospective cohort study

**DOI:** 10.1080/15592294.2025.2530618

**Published:** 2025-07-14

**Authors:** Tieshi Zhu, Yong He, Yixi Wang, Le Zhao

**Affiliations:** a Department of Medical Affairs, Agricultural Reclamation Central Hospital of Guangdong, Zhanjiang, Guangdong, China; b Department of Neurology, Zhanjiang Central Hospital, Guangdong Medical University, Zhanjiang, Guangdong, China; c Department of Neurology, Liuyang Jili Hospital, Changsha, Hunan, China; d Hospital of Stomatology, Guangdong Provincial Key Laboratory of Stomatology, Guanghua School of Stomatology, Sun Yat-sen University, Guangzhou, Guangdong, China

**Keywords:** Epigenetic clock, age acceleration, mortality, aging, NHANES

## Abstract

Epigenetic clocks have been widely applied to assess biological ageing, with Age Acceleration (AA) serving as a key metric linked to adverse health outcomes, including mortality. However, the comparative predictive value of AAs derived from different epigenetic clocks for mortality risk has not been systematically evaluated. In this retrospective cohort study based on 1,942 NHANES participants (median age 65 years; 944 women), we examined the associations between AAs from multiple epigenetic clocks and the risks of all-cause, cancer-specific, and cardiac mortality. Restricted cubic spline models were used to assess the shape of these associations, and Cox proportional hazards regression was employed to quantify risk estimates. Model performance was compared using the Akaike Information Criterion (AIC) and concordance index (C-index). Our findings revealed that only GrimAge AA and GrimAge2 AA demonstrated approximately linear and positive associations with all three mortality outcomes. Both were significantly associated with increased risks of death, and these associations were consistent across most subgroups. GrimAge and GrimAge2 AAs showed very similar performance in predicting all-cause, cancer and cardiac mortality, with only small differences in AIC values and C-index scores. These findings suggest that both GrimAge and GrimAge2 are effective epigenetic biomarkers for mortality risk prediction and may be valuable tools in future ageing-related research.

## GRAPHICAL ABSTRACT

**Figure:**
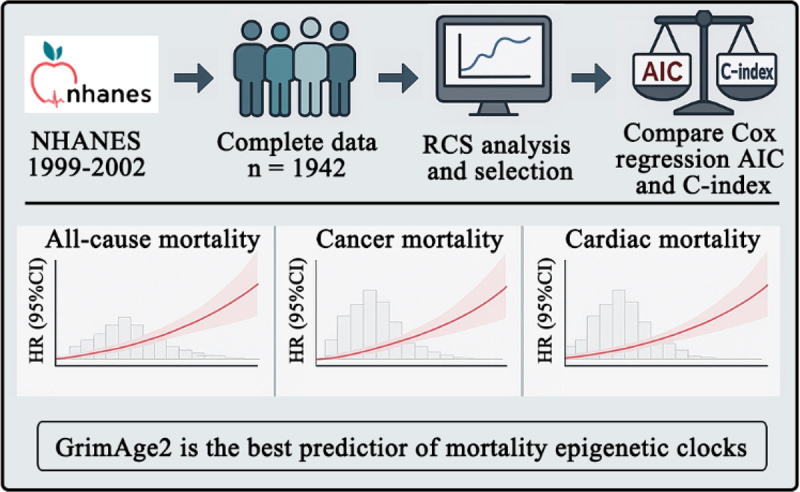

## Introduction

Ageing is a fundamental and irreversible biological process and represents a shared risk factor for numerous chronic diseases and mortality [1–4]. With the global demographic shift towards an older population, age-associated health challenges have become increasingly prominent [5,6]. However, the pace of ageing differs substantially among individuals – some experience accelerated biological ageing, whereas others demonstrate a more gradual trajectory [7–9].

To more accurately quantify individual biological ageing, researchers have developed several DNA methylation – based epigenetic clocks, including ZhangAge, GrimAge, HorvathAge, and others [10–13]. Some clocks, such as HorvathAge, HannumAge, and ZhangAge, are trained to predict chronological age, and are therefore often referred to as ‘age clocks.’ Others, such as DNAm PhenoAge, GrimAge, and GrimAge2, are trained on clinical biomarkers or mortality risk, and are commonly known as ‘mortality clocks’ [13]. These clocks enable the calculation of epigenetic age acceleration metrics, the most commonly used of which is Age Acceleration (AA). This metric reflects the degree of biological ageing by quantifying deviations from chronological age after appropriate adjustment [14–16].

Although several studies have compared the predictive performance of multiple epigenetic clocks, most have relied on linear Cox regression models without assessing the potential non-linear dose – response relationship between age acceleration and mortality risk. Therefore, further studies incorporating non-linear modelling and evaluation of model fit are still warranted [17,18]. Therefore, this study aimed to systematically assess the associations between Age Acceleration (AA), derived from several widely used epigenetic clocks, and mortality risk – including all-cause mortality, cardiac mortality, and cancer mortality – using data from the National Health and Nutrition Examination Survey (NHANES). The goal was to identify the epigenetic clock that most effectively predicts mortality risk.

## Methods

### Population

**Figure 1.**
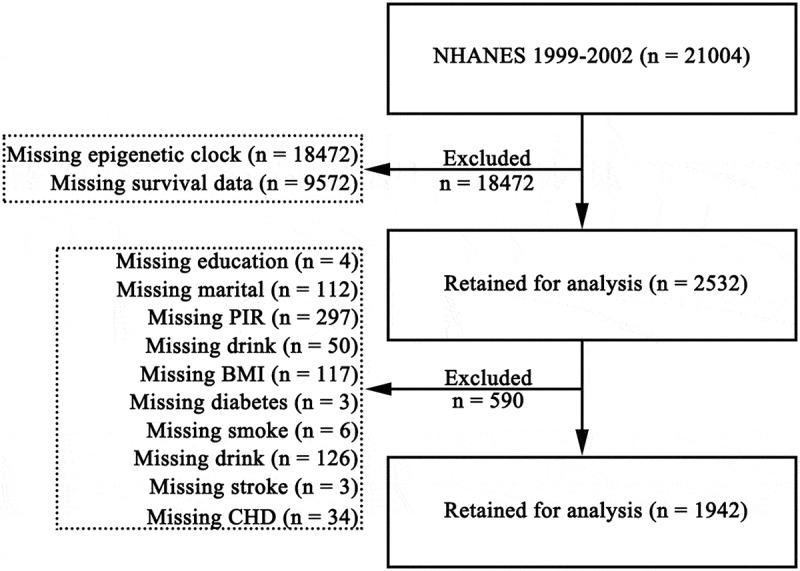
Flowchart of participants included in this study.

NHANES, National Health and Nutrition Examination Survey; PIR, poverty-to-income ratio; BMI, body mass index; CHD, coronary heart disease.

This study utilized data from the 1999–2002 cycles of NHANES, comprising 21,004 participants. Among them, 9,572 individuals lacked survival data and 18,472 lacked epigenetic clock measurements, resulting in 2,532 participants eligible for further screening. After excluding individuals with missing data on key covariates – poverty income ratio (PIR, *n* = 297), education level (*n* = 4), marital status (*n* = 112), body mass index (BMI, *n* = 117), diabetes status (*n* = 3), smoking status (*n* = 6), alcohol consumption (*n* = 126), stroke history (*n* = 3), and coronary heart disease (CHD, *n* = 34) – a total of 1,942 participants were included in the final analysis (Figure 1).

### Outcome

The endpoints of this study were all-cause mortality, cancer-specific mortality, and cardiac mortality. Mortality status was determined using the ‘MORTSTAT’ variable, and follow-up time was calculated using the ‘PERMTH_INT’ variable, both obtained from the National Death Index (NDI) – linked mortality data. Causes of death were classified based on International Classification of Diseases (ICD) codes: cardiac deaths were defined as ICD-10 codes I00–I09, I11, I13, and I20–I51; cancer deaths were defined as codes C00–C97 [18,19].

### Epigenetic clocks and AA

Epigenetic clock data in this study were derived from the 2024 NHANES release and included HorvathAge, HannumAge, SkinBloodAge, PhenoAge, GrimAge, GrimAge2, ZhangAge, LinAge, WeidnerAge, VidalBraloAge, DunedinPoAm, among others. We selected these 11 epigenetic clocks because they are among the most widely used and validated models in the epigenetic ageing literature. They represent a diverse spectrum of clock types, including those trained on chronological age (e.g., HorvathAge, HannumAge), clinical phenotypes (e.g., PhenoAge, GrimAge, GrimAge2), and population- or tissue-specific characteristics (e.g., ZhangAge, SkinBloodAge). This selection allowed for a comprehensive comparison of models with different biological underpinnings and predictive targets. DNA methylation was assessed using the Illumina Infinium MethylationEPIC BeadChip v1.0 platform. Raw IDAT image files underwent chromatic aberration correction, background subtraction, and BMIQ normalization. Data preprocessing followed the normalization pipeline and reference set developed by Horvath, resulting in a high-quality matrix of methylation beta values. Using these methylation data, the NHANES research team estimated epigenetic age for each clock based on CpG sites and regression coefficients as specified by the original developers of each algorithm. To evaluate the deviation of epigenetic age from chronological age, AA was calculated for each clock. This was done by fitting a linear regression model with epigenetic age as the dependent variable and chronological age as the independent variable; the resulting residuals were defined as AA [20]. Full details of DNA methylation biomarker computation are available in the NHANES Epigenetic Biomarkers Documentation (https://wwwn.cdc.gov/Nchs/Nhanes/DNAm/Default.aspx).

### Covariate

The variables adjusted for in this study included age, sex, race, PIR, BMI, educational attainment, marital status, cigarette smoking, alcohol intake, hypertension, diabetes, stroke, and CHD. Most of these covariates were sourced directly from the NHANES dataset. Race/ethnicity was categorized into four groups: White, Black, Mexican American, and Other (which combined Other Hispanic and Other Race – including Multi-Racial), following previous studies. Diabetes was identified based on any of the following criteria: (1) self-reported physician diagnosis; (2) current use of insulin or other glucose-lowering agents; (3) random blood glucose exceeding 11.1 mmol/L; (4) two-hour plasma glucose level above 11.1 mmol/L in an oral glucose tolerance test; or (5) haemoglobin A1c greater than 6.5% [21]. Hypertension was determined if any of the following applied: (1) a physician’s diagnosis; (2) use of antihypertensive medication; or (3) an average systolic/diastolic blood pressure reading of at least 140/90 mmHg [22]. Smoking behaviour was categorized into three groups: ‘Never’ (fewer than 100 cigarettes smoked in total), ‘Former’ (over 100 cigarettes smoked in the past, but currently not smoking), and ‘Current’ (over 100 cigarettes smoked and still smoking) [23]. Alcohol use was also divided into ‘Never’ (fewer than 12 drinks ever consumed), ‘Former’ (more than 12 drinks in a lifetime but none in the past year), and ‘Current’ (more than 12 lifetime drinks and consumption within the last year) [24].

### Statistic

Continuous variables were presented as medians with interquartile ranges (IQRs), and categorical variables were expressed as counts and percentages. Restricted cubic spline (RCS) analysis and Cox proportional hazards regression were used to evaluate the association between AA and mortality risk. Covariates included in the models were age, sex, race, BMI, PIR, education level, marital status, smoking status, alcohol consumption, hypertension, diabetes, stroke, and CHD. Variance inflation factor (VIF) analysis was used to assess multicollinearity; variables with VIF > 10 were excluded from the models, and no significant multicollinearity was detected. In subgroup analyses of Cox regression, covariates were adjusted similarly to the main models, excluding the stratification variable. A two-sided *p* value < 0.05 was considered statistically significant. Model performance was compared using the Akaike Information Criterion (AIC) and the concordance index (C-index). All statistical analyses were conducted using R software, version 4.4.1. The following R packages were used: nhanesR (version 0.9.5.2) for data import and preprocessing; dplyr (version 1.1.4) for data manipulation [25]; tableone (version 0.13.2) for baseline table generation [26]; survival (version 3.7–0) for Cox proportional hazards modelling [27]; and rcssci (version 0.4.0) for restricted cubic spline analysis [28].

Since the RCS plotting package we used does not support survey weights, and AIC values cannot be compared under complex sampling designs, our primary analyses were conducted without weighting. However, to ensure the robustness of our findings, we additionally performed weighted analyses following NHANES analytical guidelines using the survey package (version 4.4–2) [29]. Specifically, we applied the recommended weight wtdn4yr, and the corresponding results are presented in Tables S1 and S2.

## Results

### Baseline characteristics and survival outcomes

Table 1 summarizes the baseline characteristics of the 1,942 participants included in this study. The median age was 65 years, with a median PIR of 2.29 and a median BMI of 27.98. Of the participants, 48.61% were female, 42.58% identified as non-Hispanic White, 35.74% had completed high school or higher education, and 63.13% were married. Additionally, 23.33% had diabetes mellitus, 63.34% had hypertension, 14.16% were current smokers, and 53.86% reported alcohol consumption. A history of stroke and CHD was present in 4.79% and 8.19% of participants, respectively. During a median follow-up period of 208 months, 997 participants (51.34%) died, including 204 (10.50%) from cancer and 262 (13.49%) from cardiac disease (Table 1).

**Table 1.** Baseline Information and survival data.

Variables	Total (*n* = 1942)
Age (year)	65.00 (58.00, 73.00)
Female	944 (48.61)
Race	
White	827 (42.58)
Black	392 (20.19)
Mexican American	540 (27.81)
Other	183 (9.42)
PIR	2.29 (1.20, 4.31)
BMI (kg/m^2^)	27.98 (24.74, 31.53)
Education	
< High school	837 (43.10)
High school	411 (21.16)
> High school	694 (35.74)
Marital	
Married	1226 (63.13)
Never married	73 (3.76)
Separated	643 (33.11)
Smoke	
Never	901 (46.40)
Former	766 (39.44)
Current	275 (14.16)
Drink	
Never	338 (17.40)
Former	558 (28.73)
Current	1046 (53.86)
Diabetes	453 (23.33)
Hypertension	1230 (63.34)
Stroke	93 (4.79)
CHD	159 (8.19)
All-cause mortality	997 (51.34)
Cancer mortality	204 (10.50)
Cardiac mortality	262 (13.49)
Follow-up time (month)	208.00 (120.00, 225.00)
HorvathAge	66.27 (60.09, 72.72)
HannumAge	66.24 (59.88, 73.51)
SkinbloodAge	64.07 (57.06, 71.15)
PhenoAge	54.94 (47.79, 62.90)
GrimAge	65.53 (59.57, 72.42)
GrimAge2	71.49 (65.50, 77.94)
ZhangAge	66.63 (63.88, 69.37)
LinAge	56.31 (48.77, 65.23)
WeidnerAge	53.18 (46.93, 60.37)
VidalbraloAge	59.61 (55.63, 64.14)
DunedinPoAm	1.10 (1.04, 1.16)
HorvathAge Age Acceleration	−0.13 (−2.96, 2.93)
HannumAge Age Acceleration	0.09 (−2.97, 3.12)
SkinbloodAge Age Acceleration	0.08 (−2.37, 2.47)
PhenoAge Age Acceleration	0.04 (−4.11, 4.01)
GrimAge Age Acceleration	−0.80 (−3.42, 2.81)
GrimAge2 Age Acceleration	−0.65 (−3.62, 3.08)
ZhangAge Age Acceleration	0.01 (−0.75, 0.86)
LinAge Age Acceleration	−0.14 (−4.56, 4.29)
WeidnerAge Age Acceleration	−0.29 (−5.67, 5.26)
VidalbraloAge Age Acceleration	−0.18 (−3.26, 3.08)
DunedinPoAm Age Acceleration	−0.01 (−0.06, 0.05)

Data are presented as median (Q1, Q3) or n (%). Q1, 1st Quartile; Q3, 3rd Quartile; PIR, poverty-to-income ratio; BMI, body mass index; CHD, coronary heart disease.

The median values of epigenetic age across the clocks were as follows: HorvathAge, 66.27; HannumAge, 66.24; SkinBloodAge, 64.07; PhenoAge, 54.94; GrimAge, 65.53; GrimAge2, 71.49; ZhangAge, 66.63; LinAge, 56.31; WeidnerAge, 53.18; VidalBraloAge, 59.61, and DunedinPoAm, 1.10. Corresponding median AA values were: HorvathAge, −0.13; HannumAge, 0.09; SkinBloodAge, 0.08; PhenoAge, 0.04; GrimAge, −0.80; GrimAge2, −0.65; ZhangAge, 0.01; LinAge, −0.14; WeidnerAge, −0.29; VidalBraloAge, −0.18, and DunedinPoAm, −0.01 (Table 1).

### Association between AA and All-cause mortality

**Figure 2.**
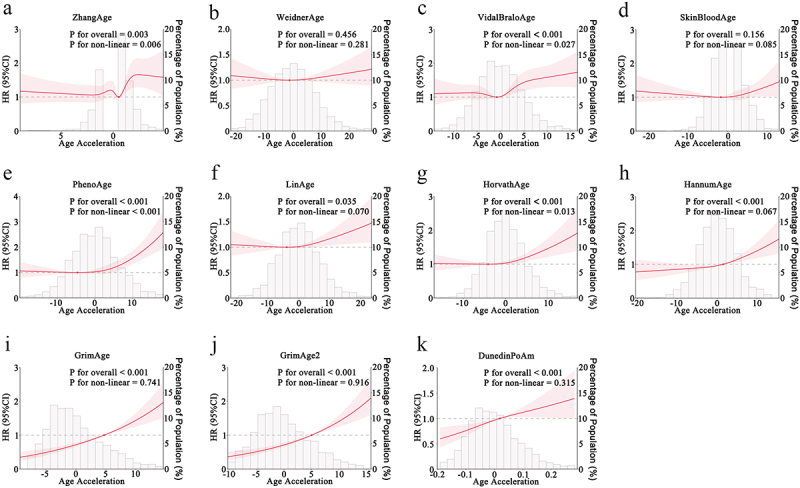
Restricted cubic spline analysis of the association between age acceleration from different epigenetic clocks and all-cause mortality.

ZhangAge AA exhibited a nonlinear association with all-cause mortality, characterized by a stable trend at lower levels, followed by an increase, then a decrease, and subsequently another increase. The elevation in mortality risk was more pronounced when ZhangAge AA exceeded zero (Figure 2a). Both WeidnerAge AA and SkinBloodAge AA demonstrated a U-shaped relationship with all-cause mortality, indicating elevated risk at both lower and higher ends of AA (Figure 2b,d).In contrast, VidalBraloAge AA, PhenoAge AA, LinAge AA, and HorvathAge AA showed an inverse L-shaped association, with mortality risk remaining relatively stable at lower AA levels and increasing as AA rose (Figure 2c, e-g). For GrimAge AA, GrimAge2 AA, and HannumAge AA, a positive linear association with all-cause mortality was observed, with risk increasing consistently at higher AA levels (Figure 2h-j). The mortality risk curves for GrimAge AA, GrimAge2 AA and DunedinPoAm closely approximated a straight line (Figure 2i-k). In addition, DNA methylation-based telomere length (DNAmTL) was inversely associated with all-cause mortality (Figure S1). Notably, while the overall trends were consistent, some confidence intervals – particularly at the extremes of the AA distribution – were relatively wide, possibly due to smaller sample sizes in those ranges. This should be considered when interpreting risk estimates at the tails of the distribution. Nevertheless, the central trends remained robust and visually coherent.

(a) ZhangAge; (b) WeidnerAge; (c) VidalBraloAge; (d) SkinBloodAge; (e) PhenoAge; (f) LinAge; (g) HorvathAge; (h) HannumAge; (i) GrimAge; (j) GrimAge2; (k) DunedinPoAm.

### Associations of selected AA with cancer and cardiac mortality

**Figure 3.**
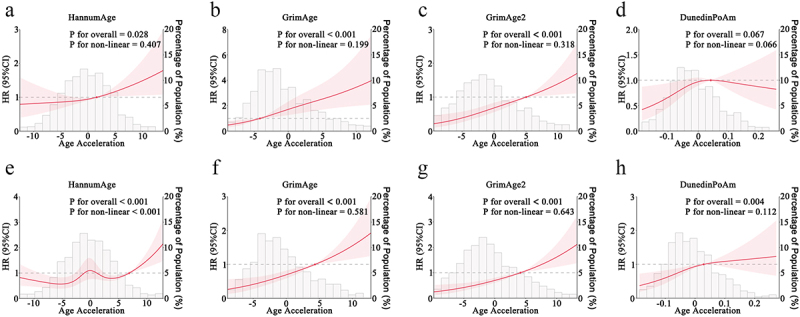
Restricted cubic spline analysis of the association between selected epigenetic age acceleration measures and cause-specific mortality.

Higher levels of GrimAge AA, GrimAge2 AA, and HannumAge AA were associated with an increased risk of cancer mortality (Figure 3a-c). DunedinPoAm showed a plateau-like association with cancer mortality (Figure 3d). The associations for GrimAge AA and GrimAge2 AA followed an approximately linear trend, with risk rising steadily as AA increased (Figure 3b,c). Similarly, GrimAge AA and GrimAge2 AA were positively associated with cardiac mortality, and their corresponding risk curves also approximated a straight line (Figure 3f,g). In contrast, the relationship between HannumAge AA and cardiac mortality followed a W-shaped pattern, indicating fluctuating risk levels across the AA spectrum (Figure 3e). DunedinPoAm is positively, but non-linearly, associated with cardiac mortality (Figure 3h). Again, although the confidence bands were wider at distribution extremes, the observed trend remains evident.

(a-d) Cancer mortality: (a) HannumAge, (b) GrimAge, (c) GrimAge2, (d) DunedinPoAm.

(e-h) Cardiac mortality: (e) HannumAge, (f) GrimAge, (g) GrimAge2, (h) DunedinPoAm.

### Comparative performance of GrimAge2 AA and GrimAge AA for mortality outcomes

Cox regression analyses, adjusted for potential confounders, demonstrated that both GrimAge AA and GrimAge2 AA were significantly associated with increased risks of all-cause, cardiac, and cancer mortality. Specifically, each 1-unit increase in GrimAge AA was associated with higher risk of all-cause mortality (HR, 1.07; 95% CI, 1.06–1.09; *p* < .01), cardiac mortality (HR, 1.09; 95% CI, 1.05–1.13; *p* < .01), and cancer mortality (HR, 1.09; 95% CI, 1.05–1.13; *p* < .01). Similarly, GrimAge2 AA showed consistent associations with all-cause mortality (HR, 1.07; 95% CI, 1.05–1.09; *p* < .01), cardiac mortality (HR, 1.09; 95% CI, 1.06–1.13; *p* < .01), and cancer mortality (HR, 1.09; 95% CI, 1.05–1.13; *p* < .01) (Tables 2 and 3). Subgroup analyses further supported these findings. GrimAge AA was significantly associated with all-cause mortality across all subgroups, and with cardiac and cancer mortality in most subgroups (Table 2). GrimAge2 AA was significantly associated with all three mortality outcomes across most subgroups (Table 3). After applying survey weights, GrimAge AA and GrimAge2 AA remained significantly associated with all-cause mortality, cardiac mortality, and cancer mortality, as well as in most subgroup analyses (Tables S1 and S2). Furthermore, when participants aged ≥85 years were excluded, GrimAge AA and GrimAge2 AA remained significantly associated with all three mortality outcomes, and the results were largely consistent with the main analysis (Table S3).

**Table 2.** Association of GrimAge age Acceleration with all-cause, cardiac, and cancer mortality: cox regression and subgroup analyses.

	All-cause mortality		Cardiac mortality		Cancer mortality	
	HR(95%CI)	*P*	HR(95%CI)	*P*	HR(95%CI)	*P*
Overall	1.07(1.06,1.09)	< 0.01	1.09(1.05,1.13)	< 0.01	1.09(1.05,1.13)	< 0.01
Age						
< 65	1.06(1.03,1.10)	< 0.01	1.1(1.02,1.17)	0.01	1.02(0.96, 1.09)	0.52
≥65	1.08(1.06,1.10)	< 0.01	1.09(1.05,1.14)	< 0.01	1.13(1.07, 1.18)	< 0.01
Gender						
Female	1.1(1.07,1.13)	< 0.01	1.07(1.02,1.12)	< 0.01	1.14(1.06,1.22)	< 0.01
Male	1.06(1.04,1.09)	< 0.01	1.14(1.07, 1.21)	< 0.01	1.07(1.02,1.12)	0.01
BMI						
< 25	1.07(1.04,1.11)	< 0.01	1.06(0.99,1.15)	0.1	1.13(1.04, 1.24)	< 0.01
25–30	1.06(1.03,1.10)	< 0.01	1.11(1.04, 1.17)	< 0.01	1.08(1.02,1.16)	0.02
≥30	1.07(1.04,1.11)	< 0.01	1.09(1.02, 1.17)	0.01	1.08(1.01, 1.16)	0.03
PIR						
0–1	1.09(1.05,1.14)	< 0.01	1.14(1.03, 1.26)	0.01	1.15(1.04, 1.26)	< 0.01
1.1–3.0	1.07(1.04,1.09)	< 0.01	1.1(1.04,1.16)	< 0.01	1.11(1.04,1.18)	< 0.01
> 3.0	1.09(1.06,1.13)	< 0.01	1.12(1.04, 1.20)	< 0.01	1.09(1.02, 1.17)	0.02
Race						
White	1.1(1.07,1.13)	< 0.01	1.13(1.07, 1.20)	< 0.01	1.18(1.10, 1.26)	< 0.01
Black	1.07(1.03,1.11)	< 0.01	1.11(1.03, 1.20)	0.01	1.1(1.01, 1.19)	0.02
Mexican American	1.05(1.01,1.09)	0.01	1.05(0.97, 1.14)	0.23	1.04(0.96, 1.12)	0.38
Other	1.07(1.01,1.14)	0.03	1.05(0.75, 1.47)	0.76	1.09(0.93, 1.28)	0.27
Diabetes						
Yes	1.07(1.03,1.11)	< 0.01	1.05(0.98, 1.13)	0.13	1.13(1.03, 1.24)	0.01
No	1.07(1.05,1.09)	< 0.01	1.1(1.05,1.15)	< 0.01	1.09(1.04,1.13)	< 0.01
Hypertension						
Yes	1.08(1.06,1.10)	< 0.01	1.09(1.05,1.14)	< 0.01	1.12(1.07,1.18)	< 0.01
No	1.06(1.02,1.10)	< 0.01	1.12(1.03,1.22)	0.01	1.04(0.98,1.11)	0.20

BMI, body mass index; PIR, poverty-to-income ratio.

**Table 3.** Association of GrimAge2 age Acceleration with all-cause, cardiac, and cancer mortality: cox regression and subgroup analyses.

	All-cause mortality		Cardiac mortality		Cancer mortality	
	HR(95%CI)	*P*	HR(95%CI)	*P*	HR(95%CI)	*P*
Overall	1.07(1.05,1.09)	< 0.01	1.09(1.06,1.13)	< 0.01	1.09(1.05,1.13)	< 0.01
Age						
< 65	1.06(1.03,1.09)	< 0.01	1.1(1.04,1.17)	< 0.01	1.02(0.96, 1.08)	0.45
≥65	1.07(1.05,1.09)	< 0.01	1.09(1.05,1.14)	< 0.01	1.12(1.07, 1.17)	< 0.01
Gender						
Female	1.09(1.06,1.12)	< 0.01	1.09(1.04,1.13)	< 0.01	1.13(1.07,1.20)	< 0.01
Male	1.06(1.04,1.08)	< 0.01	1.12(1.06,1.19)	< 0.01	1.07(1.02,1.11)	< 0.01
BMI						
< 25	1.06(1.03,1.10)	< 0.01	1.07(1.00,1.14)	0.04	1.13(1.04, 1.22)	< 0.01
25–30	1.06(1.04,1.09)	< 0.01	1.11(1.05,1.18)	< 0.01	1.08(1.02,1.15)	0.01
≥30	1.07(1.04,1.10)	< 0.01	1.09(1.02,1.16)	0.01	1.08(1.01, 1.15)	0.02
PIR						
0–1	1.09(1.04,1.13)	< 0.01	1.14(1.04,1.25)	0.01	1.14(1.04, 1.24)	< 0.01
1.1–3.0	1.06(1.04,1.09)	< 0.01	1.1(1.05, 1.15)	< 0.01	1.09(1.03,1.15)	< 0.01
> 3.0	1.09(1.06,1.12)	< 0.01	1.12(1.05,1.19)	< 0.01	1.09(1.02, 1.16)	0.01
Race						
White	1.09(1.06,1.12)	< 0.01	1.12(1.06,1.18)	< 0.01	1.15(1.09, 1.22)	< 0.01
Black	1.07(1.04,1.11)	< 0.01	1.12(1.05,1.19)	< 0.01	1.12(1.03, 1.21)	0.01
Mexican American	1.04(1.01,1.08)	0.01	1.06(0.98,1.14)	0.17	1.05(0.97,1.13)	0.23
Other	1.06(1.00,1.12)	0.06	1.08(0.85,1.37)	0.53	1.02(0.87, 1.19)	0.81
Diabetes						
Yes	1.06(1.03,1.10)	< 0.01	1.06(1.00,1.13)	0.04	1.1(1.01, 1.19)	0.03
No	1.07(1.05,1.09)	< 0.01	1.11(1.06,1.15)	< 0.01	1.09(1.05,1.14)	< 0.01
Hypertension						
Yes	1.07(1.06,1.09)	< 0.01	1.09(1.05,1.13)	< 0.01	1.11(1.06,1.16)	< 0.01
No	1.06(1.02,1.09)	< 0.01	1.13(1.05,1.22)	< 0.01	1.05(0.99,1.12)	0.09

BMI, body mass index; PIR, poverty-to-income ratio.

In terms of model performance, Cox models incorporating GrimAge2 AA yielded slightly lower AIC values than those using GrimAge AA across all-cause, cardiac, and cancer mortality, suggesting marginally better model fit. The C-index values for GrimAge2 AA were also slightly higher for all-cause and cardiac mortality, while the C-index for cancer mortality was marginally lower than that of GrimAge AA (Table 4). These findings indicate that the predictive performance of GrimAge and GrimAge2 AAs is largely comparable.

**Table 4.** Comparison of GrimAge and GrimAge2 age acceleration in predicting all-cause, cancer, and cardiovascular mortality.

	Age Acceleration	HR(95%CI)	AIC	C_index
All-cause mortality	GrimAge	1.07(1.06,1.09)	13347.66	0.779
	GrimAge2	1.07(1.05,1.09)	13340.84	0.780
Cancer mortality	GrimAge	1.09(1.05,1.13)	2596.87	0.782
	GrimAge2	1.09(1.05,1.13)	2593.60	0.781
Cardiac mortality	GrimAge	1.09(1.05,1.13)	3104.82	0.866
	GrimAge2	1.09(1.06,1.13)	3097.34	0.867

## Discussion

In this study, using data from the 1999–2002 cycles of NHANES, we found that both GrimAge AA and GrimAge2 AA were significantly and positively associated with all-cause, cancer-specific, and cardiac mortality. These associations were approximately linear across the range of AA, a pattern not observed with other epigenetic clocks evaluated in this analysis. Comparative assessment of model performance showed minimal differences between the two clocks, with GrimAge2 AA yielding slightly lower AIC values and marginally higher C-index scores for all-cause and cardiac mortality. These findings indicate that both GrimAge and GrimAge2 are effective and reliable predictors of mortality risk in population-based settings.

Many epigenetic clocks have demonstrated the ability to capture aspects of biological ageing, and the derived AA metric offers an intuitive measure of age-related deviation. As a result, AA is frequently used to investigate associations with mortality and disease risk [30,31]. However, despite the multidimensional nature of ageing, we propose that mortality remains its most fundamental and clinically relevant endpoint [32]. If an epigenetic clock fails to accurately predict mortality, its utility as a biomarker of ageing may be limited. From a conceptual standpoint, individuals undergoing accelerated biological ageing should exhibit a substantially higher risk of death, whereas those ageing more slowly should experience reduced mortality risk [33,34]. In fact, the study by Mendy et al. also utilized NHANES data and reported that GrimAge2 outperformed other epigenetic clocks in predicting mortality. However, their study did not perform RCS model, subgroup analyses, or an evaluation of GrimAge itself [18]. In contrast, our study not only included these additional analyses but also showed that GrimAge and GrimAge2 had similarly strong predictive performance for mortality. Given that most of the clocks included in this study were first-generation clocks trained to predict chronological age, it is not surprising that GrimAge and GrimAge2—which were explicitly developed to predict mortality – demonstrated more consistent and approximately linear associations with mortality outcomes. Although linear associations are more interpretable, non-linear patterns may also have biological relevance, as suggested by U-shaped relationships between telomere length and cancer and inverse associations between epigenetic age and cancer risk [35,36].

In this study, VidalBraloAge AA, PhenoAge AA, LinAge AA, and HorvathAge AA exhibited inverse L-shaped associations with all-cause mortality, with relatively stable risk at lower AA levels and increased risk at higher levels. This pattern suggests that these clocks may be more suitable for identifying mortality risk in individuals with pronounced biological ageing, but may have limited predictive utility in populations with lower AA.In contrast, WeidnerAge AA and SkinBloodAge AA demonstrated U-shaped relationships with all-cause mortality, indicating increased risk at both low and high AA levels. This is biologically implausible and suggests that these clocks may be less reliable indicators of mortality risk. ZhangAge AA also demonstrated substantial variability and an inconsistent relationship with all-cause mortality, further limiting its applicability for mortality prediction. HannumAge AA was positively associated with both all-cause and cancer-related mortality, but not with cardiac mortality, suggesting moderate predictive capacity. However, it may not represent the optimal epigenetic clock due to its limited consistency across endpoints. By contrast, GrimAge AA and GrimAge2 AA were significantly and positively associated with all-cause, cancer, and cardiac mortality in a near-linear manner. These associations remained robust across most subgroup analyses, underscoring their strong and stable predictive performance. Based on lower AIC values and higher C-index values in Cox regression models, Although GrimAge2 AA and GrimAge AA showed largely comparable predictive performance, GrimAge2 exhibited slightly lower AIC values, consistent with the findings reported by [37]. The superior performance of GrimAge2 AA may be attributed to its updated training process, which directly targets time-to-death using elastic net regression, and incorporates DNA methylation surrogates for mortality-related plasma proteins and smoking history. In addition, GrimAge2 includes two new components – epigenetic predictors of CRP and HbA1c – which may further enhance its relevance to ageing and mortality. Notably, formal model performance metrics were compared only between GrimAge2 and GrimAge; comparisons with other clocks were based on visual interpretation of spline curves and biological plausibility. In addition to its predictive performance, GrimAge2 offers practical advantages over clocks such as HorvathAge, SkinBloodAge, and ZhangAge, as it relies solely on whole blood samples without the need for additional tissues [10,11,37,38]. However, it is important to acknowledge that incorporating multiple tissue types may capture a broader spectrum of ageing-related changes and potentially enhance accuracy. Therefore, future efforts to refine and optimize multi-tissue clocks – such as HorvathAge, SkinBloodAge, and ZhangAge – may also yield improved predictive value, warranting further investigation.

This study not only confirms the robust and consistent predictive performance of the GrimAge family of epigenetic clocks across multiple mortality outcomes, but also proposes a unified and practical criterion for evaluating the utility of epigenetic clocks – namely, whether they demonstrate an approximately linear association with both all-cause and cause-specific mortality risk. Our findings highlight that, among the many available epigenetic clocks, only a limited number exhibit broad applicability and stable predictive capacity. GrimAge and GrimAge2, in particular, showed strong and consistent associations with mortality risk, suggesting their utility in future research on epigenetic ageing and biomarker development. Further investigations are warranted to assess the relevance of these clocks to other ageing-related outcomes, such as functional decline, quality of life, and incident cardiac events. Moreover, validation in diverse populations and geographic settings is essential to expand their potential applications in clinical practice and geriatric health management.

This study has several limitations. First, the analysis was restricted to adults aged 50 years and older from the 1999–2002 NHANES cycles, which may limit the generalizability of the findings to younger populations. Second, epigenetic age was estimated from a single time-point measurement of DNA methylation, precluding assessment of longitudinal changes in the ageing trajectory. Third, cause-of-death information was obtained from the NDI, and potential misclassification in the coding of specific causes of death cannot be ruled out. Fourth, several epigenetic clock models included CpG sites that were not directly available in the NHANES dataset and required imputation, which may have introduced estimation error and affected the precision of some clocks. Finally, although mortality was the primary outcome in this study, other important ageing-related outcomes – such as physical and cognitive function, disability, and quality of life – were not included. As the study population was derived from a U.S.-based cohort, caution is warranted when extrapolating these findings to populations in other regions or with different demographic characteristics. Lastly, given the number of statistical tests conducted across multiple epigenetic clocks and mortality outcomes, the possibility of type I error inflation due to multiple comparisons cannot be excluded. Although we did not apply formal multiple testing corrections (e.g., Bonferroni or FDR), most of our key findings were highly significant and supported by consistency across model performance metrics. Nevertheless, we recommend cautious interpretation of associations with borderline significance.

## Conclusion

Among multiple epigenetic clocks AA, only GrimAge AA and GrimAge2 AA showed stable and approximately linear associations with all-cause, cardiac, and cancer mortality. Although GrimAge2 AA showed slightly better model fit in some outcomes, the overall differences between the two were minimal. These findings suggest that both GrimAge and GrimAge2 AAs are practical and reliable biomarkers for biological ageing and mortality risk.

## Supplementary Material

table S1.docx

table S2.docx

Table S3.docx

Fig S1.tif

## Data Availability

The data for this study were obtained from the NHANES database, publicly accessible at https://www.cdc.gov/nchs/nhanes/.
