# Supplementary material for: GrimAge and GrimAge2 Age Acceleration effectively predict mortality risk: a retrospective cohort study
Source: Epigenetics. 2025 Jul 14;20(1):2530618. doi: 10.1080/15592294.2025.2530618 (PMC12269703; doi:10.1080/15592294.2025.2530618)
Supplement: table S2.docx [file KEPI_A_2530618_SM2105.docx]

|  | **All-cause mortality** | | **Cardiac mortality** | | **Cancer mortality** | |
| --- | --- | --- | --- | --- | --- | --- |
|  | **HR(95%CI)** | ***P*** | **HR(95%CI)** | ***P*** | **HR(95%CI)** | ***P*** |
| **Overall** | 1.08(1.06,1.11) | <0.01 | 1.12(1.08,1.17) | <0.01 | 1.11(1.05,1.18) | <0.01 |
| **Age** |  |  |  |  |  |  |
| **<65** | 1.07(1.02,1.12) | <0.01 | 1.09(1.04,1.15) | <0.01 | 1.05(0.96, 1.15) | 0.27 |
| **>=65** | 1.09(1.06,1.13) | <0.01 | 1.14(1.07,1.20) | <0.01 | 1.17(1.10,1.26) | <0.01 |
| **Gender** |  |  |  |  |  |  |
| **Female** | 1.09(1.06,1.13) | <0.01 | 1.16(1.07,1.26) | <0.01 | 1.11(1.02, 1.21) | 0.01 |
| **Male** | 1.08(1.04,1.11) | <0.01 | 1.11(1.05,1.16) | <0.01 | 1.11(1.05,1.17) | <0.01 |
| **BMI** |  |  |  |  |  |  |
| **<25** | 1.12(1.06,1.19) | <0.01 | 1.14(1.06,1.23) | <0.01 | 1.20( 1.06, 1.35) | <0.01 |
| **25-30** | 1.07(1.03,1.11) | <0.01 | 1.09(1.02,1.18) | 0.02 | 1.09(0.98,1.21) | 0.12 |
| **>=30** | 1.06(1.03,1.10) | <0.01 | 1.12(1.07,1.18) | <0.01 | 1.10(1.01, 1.20) | 0.03 |
| **PIR** |  |  |  |  |  |  |
| **0-1** | 1.11(1.04,1.18) | <0.01 | 1.15(1.03,1.28) | 0.01 | 1.10(0.95, 1.26) | 0.20 |
| **1.1-3.0** | 1.08(1.04,1.11) | <0.01 | 1.13(1.06,1.20) | <0.01 | 1.12(1.02, 1.23) | 0.01 |
| **>3.0** | 1.09(1.06,1.13) | <0.01 | 1.12(1.07,1.18) | <0.01 | 1.10(1.04,1.18) | <0.01 |
| **Race** |  |  |  |  |  |  |
| **White** | 1.09(1.06,1.12) | <0.01 | 1.13(1.07,1.19) | <0.01 | 1.13(1.06,1.21) | <0.01 |
| **Black** | 1.08(1.05,1.12) | <0.01 | 1.12(1.03,1.22) | 0.01 | 1.11(1.05, 1.18) | <0.01 |
| **Mexican American** | 1.04(0.99,1.08) | 0.13 | 1.03(0.94,1.12) | 0.56 | 1.04(0.98,1.12) | 0.20 |
| **Other** | 1.03(0.99,1.08) | 0.15 | 1.11(0.95,1.29) | 0.20 | 0.99( 0.81, 1.21) | 0.92 |
| **Diabetes** |  |  |  |  |  |  |
| **Yes** | 1.08(1.03,1.13) | <0.01 | 1.09(1.00,1.20) | 0.05 | 1.14(0.95, 1.38) | 0.16 |
| **No** | 1.08(1.05,1.11) | <0.01 | 1.11(1.07,1.17) | <0.01 | 1.11(1.05,1.17) | <0.01 |
| **Hypertension** |  |  |  |  |  |  |
| **Yes** | 1.08(1.05,1.11) | <0.01 | 1.11(1.06,1.16) | <0.01 | 1.12(1.05,1.21) | <0.01 |
| **No** | 1.08(1.04,1.13) | <0.01 | 1.15(1.06,1.24) | <0.01 | 1.09(1.02, 1.17) | 0.01 |

Table S2. Survey-Weighted Cox Regression and Subgroup Analyses of GrimAge2 Age Acceleration With All-Cause, Cardiac, and Cancer Mortality

BMI, body mass index; PIR, poverty-to-income ratio.
