# Supplementary material for: GrimAge and GrimAge2 Age Acceleration effectively predict mortality risk: a retrospective cohort study
Source: Epigenetics. 2025 Jul 14;20(1):2530618. doi: 10.1080/15592294.2025.2530618 (PMC12269703; doi:10.1080/15592294.2025.2530618)
Supplement: Table S3.docx [file KEPI_A_2530618_SM2104.docx]

|  | Age Acceleration | HR(95%CI) | *P* |
| --- | --- | --- | --- |
| All-cause mortality | GrimAge | 1.07(1.06,1.09) | <0.01 |
|  | GrimAge2 | 1.07(1.05,1.09) | <0.01 |
| Cancer mortality | GrimAge | 1.09(1.05,1.14) | <0.01 |
|  | GrimAge2 | 1.09(1.05,1.13) | <0.01 |
| Cardiac mortality | GrimAge | 1.10(1.06,1.14) | <0.01 |
|  | GrimAge2 | 1.10(1.06,1.13) | <0.01 |

Table S3. Associations Between GrimAge and GrimAge2 Age Acceleration and Mortality Risk After Excluding Participants Aged ≥85 Years
