# Supplementary figures and images for: GrimAge and GrimAge2 Age Acceleration effectively predict mortality risk: a retrospective cohort study
Source: Epigenetics. 2025 Jul 14;20(1):2530618. doi: 10.1080/15592294.2025.2530618 (PMC12269703; doi:10.1080/15592294.2025.2530618)

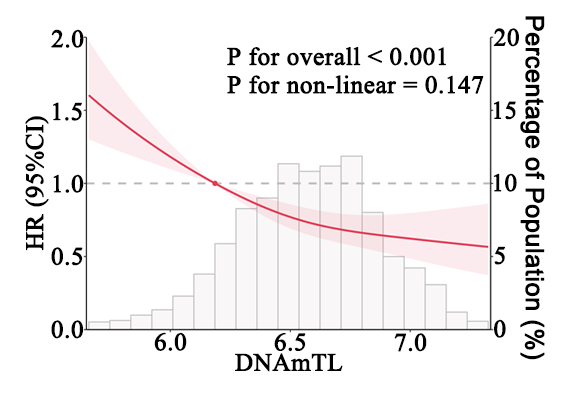

Supplement: Fig S1.tif [file KEPI_A_2530618_SM2103.tif]
